# Supplementary material for: Essential Oils of Five Syzygium Species Growing Wild in Vietnam: Chemical Compositions and Antimicrobial and Mosquito Larvicidal Potentials
Source: Molecules. 2023 Nov 9;28(22):7505. doi: 10.3390/molecules28227505 (PMC10672915; doi:10.3390/molecules28227505)
Supplement: Supplementary file 1 [file molecules-28-07505-s001.zip › molecules-2661957-supplementary.pdf]

molecules

MDPI

Article

Essential oils of five *Syzygium* species growing wild in Vietnam: Chemical compositions, antimicrobial and mosquito larvicidal potentials

Le Thi Huong [1], Nguyen Huy Hung [2], Nguyen Ngoc Linh [3], Ty Viet Pham [4], Do Ngoc Dai [5], Nguyen Quang Hop [6], William N. Setzer [7,8], Ninh The Son [9,*], Wilfried Andlauer [10], Wolfram Manuel Brück [10,*]

[1] School of Natural Science Education, Vinh University, 182 Le Duan, Vinh City, Nghe An 43000, Vietnam; lehuong223@gmail.com
[2] Center for Advanced Chemistry, Institute of Research and Development, Duy Tan University, 03 Quang Trung, Da Nang 50000, Vietnam; nguyenhuyhung@duytan.edu.vn
[3] Faculty of Pharmacy, Thanh Do University, Kim Chung, Hoai Duc, Hanoi 10000, Vietnam; nnlinh@thanhdouni.edu.vn
[4] Faculty of Chemistry, University of Education, Hue University, 34 Le Loi, Hue 530000, Vietnam; phamvietty@hueuni.edu.vn
[5] Faculty of Agriculture, Forestry and Fishery, Nghe An College of Economics, 51-Ly Tu Trong, Vinh City, Nghe An 43000, Vietnam; daidn23@gmail.com
[6] Faculty of Chemistry, Hanoi Pedagogical University 2 (HPU2), 32 Nguyen Van Linh, Xuan Hoa, Phuc Yen, Vinh Phuc 15000, Vietnam; nguyenquanghop@hpu2.edu.vn
[7] Aromatic Plant Research Center, 230 N 1200 E, Suite 100, Lehi, UT 84043, USA; setzerw@uah.edu
[8] Department of Chemistry, University of Alabama in Huntsville, 301 Sparkman Dr NW, Huntsville, AL 35899, USA; setzerw@uah.edu
[9] Institute of Chemistry, Vietnam Academy of Science and Technology (VAST), 18 Hoang Quoc Viet, Caugiay, Hanoi 10000, Vietnam; yamantson@gmail.com
[10] Institute of Life Technologies, University of Applied Sciences and Arts Western Switzerland Valais, Rue de l'Industrie 19, 1950 Sion, Switzerland; wilfried.andlauer@hevs.ch and wolfram.bruck@hevs.ch
* Correspondence: NTS: yamantson@gmail.com and WMB: wolfram.bruck@hevs.ch; Tel.: +41-58-606-86-64

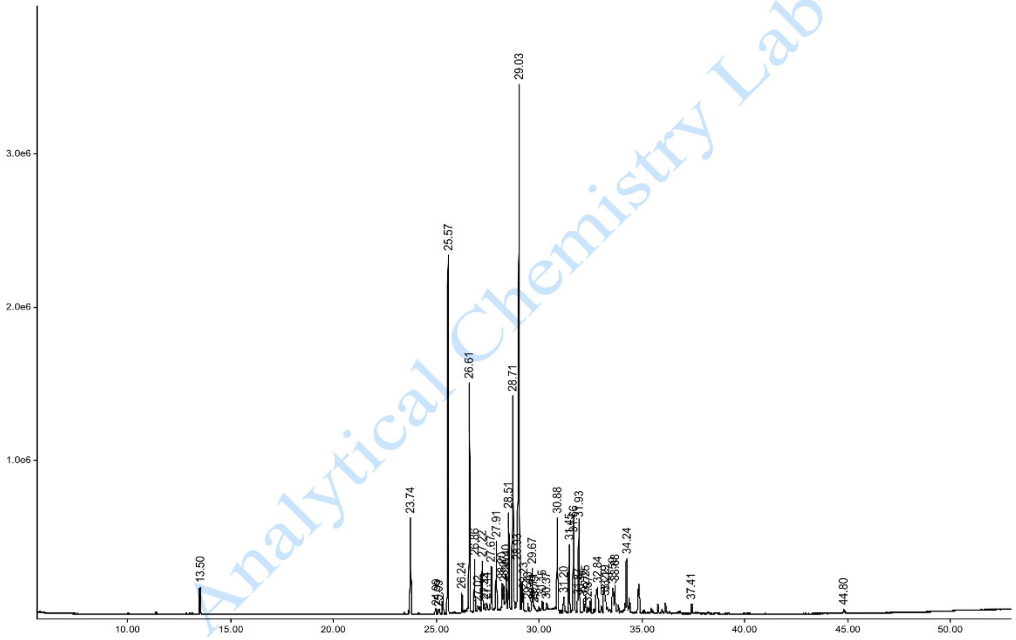

Analytical Chemistry Lab

Figure S1. GC chromatogram of *Syzygium levinei* leaf essential oil.

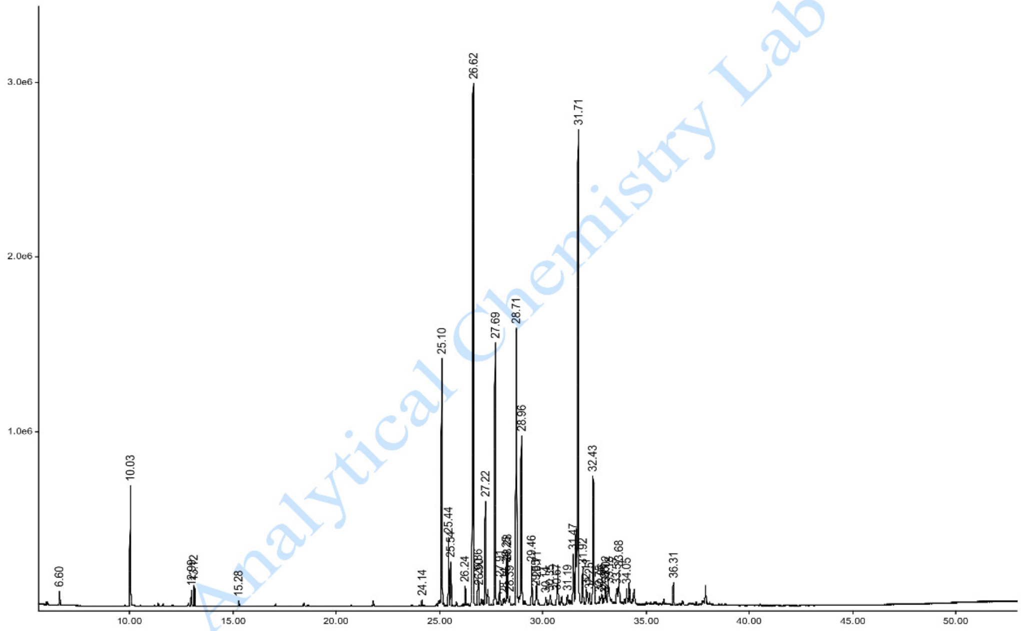

Figure S2. GC chromatogram of *Syzygium acuminatissimum* leaf essential oil.

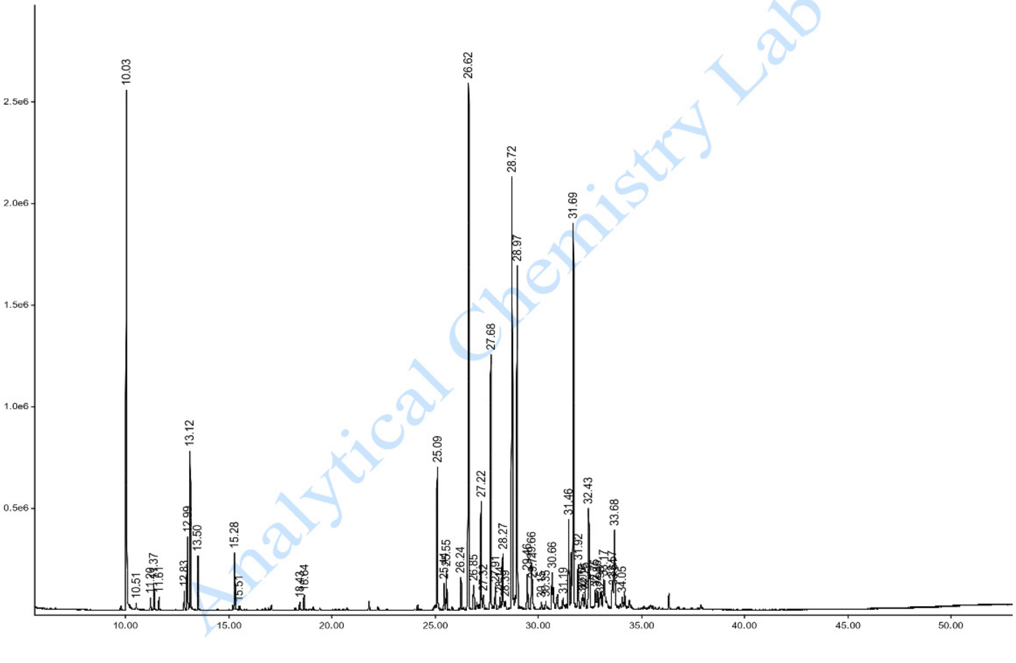

Figure S3. GC chromatogram of *Syzygium acuminatissimum* fruit essential oil.

Analytical Chemistry Lab

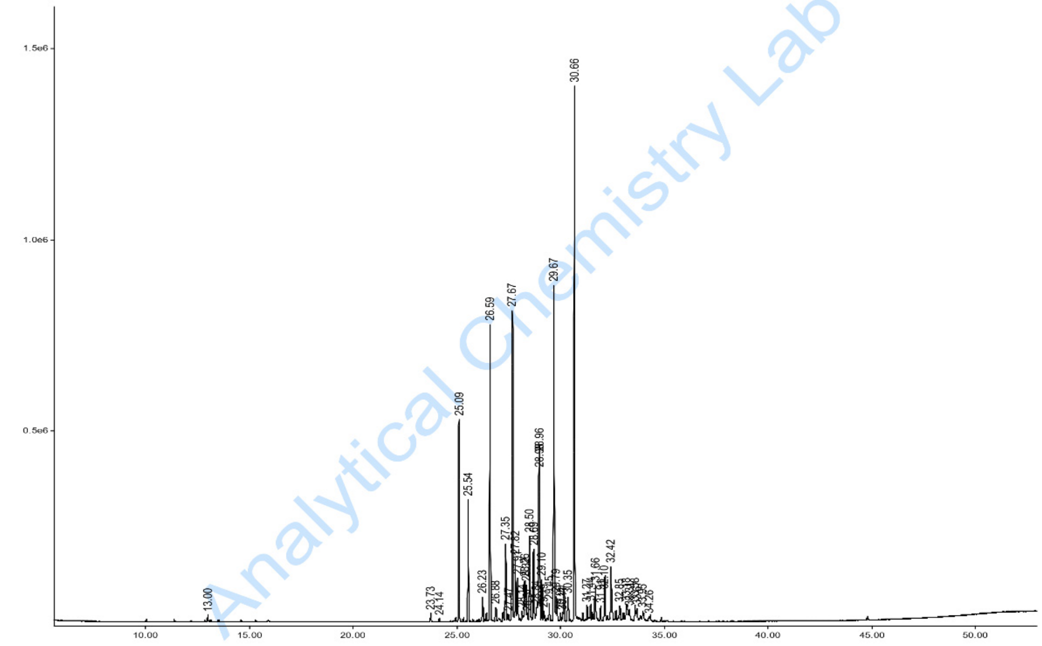

Analytical Chemistry Lab

Figure S4. GC chromatogram of *Syzygium vestitum* leaf essential oil.

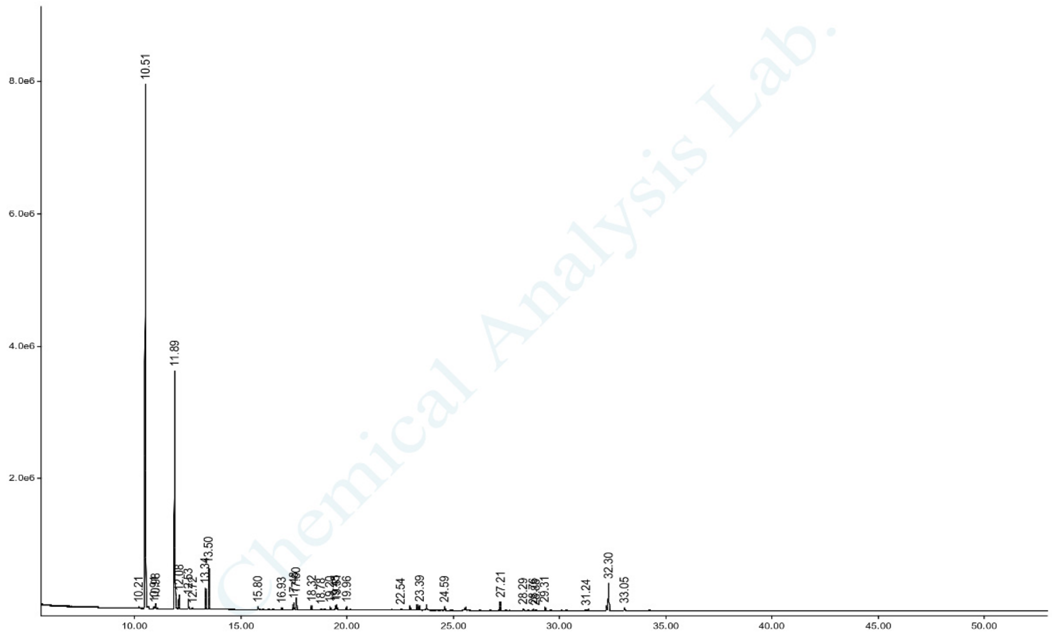

Chemical Analysis Lab.

Figure S5. GC chromatogram of *Syzygium cumini* leaf essential oil.

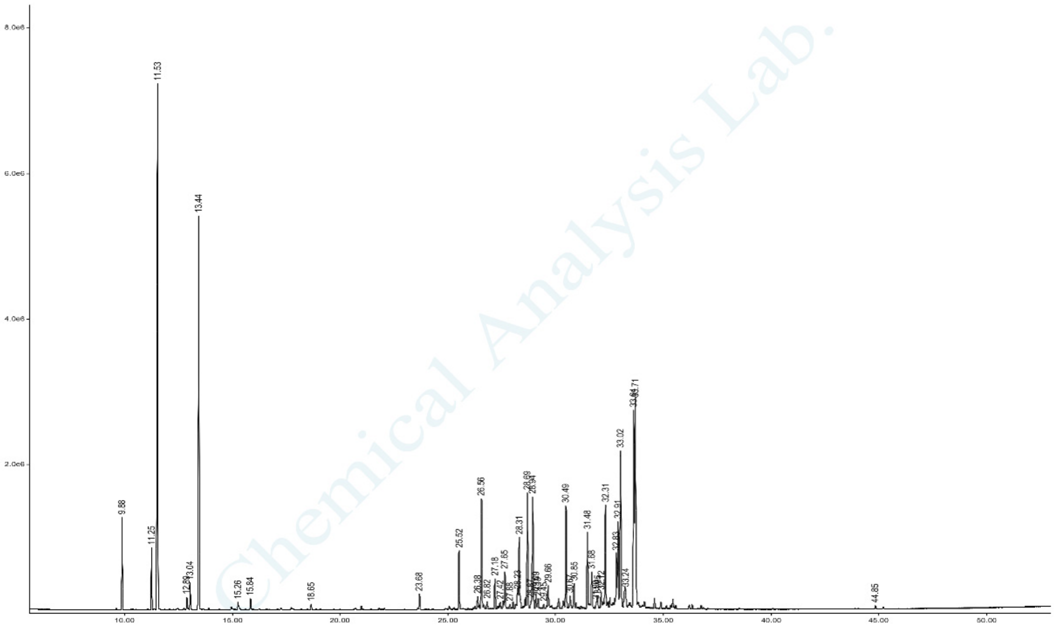
Chemical Analysis Lab.

Figure S6. GC chromatogram of *Syzygium buxifolium* leaf essential oil.
